# Remarks on the History and Use of Tobacco

**Published:** 1811-03

**Authors:** 


### *To the Editors of the Medical and Physical Journal.*

### *Remarks on the History and Use of Tobacco.*

(Concluded from page 140.)

TOBACCO has been considered as concentrating in itself the powers of every remedy. It has been employed topically to all sorts of wounds, ulcers, and herpetic diseases: and when taken internally, the most violent morbid derangements were once believed to submit to its medicinal virtues. The same *Castor Duranti* who compared its introduction into Italy with the promulgation of the blessings of religion, has given a poetical catalogue of the complaints it cures.

"Nomine quæ Sanctæ Crucis herba vocatur, ocellis

Subvenit, & sanat plagas, et vulnera jungit,

Discutit & strumas, cancrum, cancrosaque sanat

Ulcera, & ambustis prodest, scabiemque repellit;

Discutit et morbum cui cessit ab impete nomen,

Calefacit & siccat, stringit, mundatque, resolvit,

Et dentem & ventris mulcet capitisque dolores;

Subvenit antiquæ tussi, stomacoque rigenti

Renibus & spleni confert, ultroque, venena

Dira sagittarum domat, ictibus omnibus atris

Hæc eadem prodest: gingivis proficit atque

Conciliat somnum; nuda ossaque carne revestit:

Thoracis vitiis prodest, pulmonis itemque,

Quæ duo sic præstat non ulla potentior herba*.

---

* The herb which beareth *Santa Croces* name,

Sore eyes relieves, and healeth wounds: The same

 Discusses

This copious list contains but a moiety of the virtues that the gratitude or the credulity of mankind bestowed on this alien, from a, then, new found world.

An accidental, or an empirical application of it, seems first to have raised its reputation in Europe as a cure for wounds, ulcers, and cutaneous diseases. The history of this is remarkable enough. A relation of a page of the *M. Nicot*, before mentioned as having afforded the generic name to this family of plants, had a creeping ulceration upon the cheek, which seems to have been *noli me tangere* or *herpes exedens*. It had spread over the cheek, seized the nose, and corroded its alæ. By an application of the green leaf and juice of the tobacco, which Nicot had then growing in his garden at Lisbon, this corroding ulcer was cured in twelve days. This occasioned its application to be extended. Wounds were suddenly healed* by it; tetters were removed;

---

Discusses the king's evil, and removes
Cancers and boils: a remedy it proves
For burns and scalds, repels the nauseous itch,
And straight recovers from convulsion fits.
It cleanses, dries, binds up, and maketh warm:
The head-ach, tooth-ach, cholic, like a charm
It eases soon; an antient cough relieves,
And to the reins, the milt, and stomach, gives
Quick riddance from the pain that each endures:
Next the dire wounds of poisoned arrows cures;
All bruises heals, and when the gums are sore,
It makes them sound aad healthy as before.
Sleep it procures, our anxious sorrow lays,
And with new flesh the naked bone arrays.
No herb has greater power to rectify
All the disorders in the breast that lie,
Or in the lungs.

Mr. des Maizeaux is less an elegant than a faithful translator: The latin muse of the Italian physician makes a homely appearance in her English vestment. Castor Duranti resided at Rome, where he was esteemed to be a great physician and agreeable poet: Pope Sixtus the 5th held him in high consideration. He died at Viterbo in 1590, and left several works on medicine and botany in Latin and Italian. His work, Il Tesoro della sanita, nel quale si da el modo di conserver la sanita e prolongar la vita, e si tratta della natura di cibi, e dei rimedi, e dei nocumenti loro. 4to. Roma, 1589, may possibly state more particularly the grounds of his attachment to the Nicotiana. Perhaps, when he lavished his praises on the plant, he intended, with courtly dexterity, to flatter the cardinal who imported the inestimable treasure.

* There is an interesting circumstance connected with this sudden healing of wounds by the use of Tobacco. When enumerating the virtues and strumous tumours discussed. So said the oral traditions, and even the written records of that period. Succeeding times have not justified the panegyric. Modern surgery never has recourse, with these views, to any of the species of *Nicotiana*. It is not certain, however, that a fotus, cataplasm, or even an unguent prepared with the *N. Tabaccum*, might not be employed, with advantage, in some cases of indolently painful tumours, and scrofulous ulcerations. In cases of local spasm, perhaps in Trismus and Hydrophobia, they might be had recourse to as an auxiliary at least. In those obstructions of the canal of the urethra called strictures, it appears that bougies medicated with Tobacco* have been eminently useful.

---

tues of this plant, old Gerarde says, " I doe make hereof an excellente balsame to cure deepe wounds and punctures, made by some narrow sharpe pointed weapon. Which balsame doth bring up the flesh from the bottome verie speedily, *and also heale simple cuts in the flesh according to the* first intention, *that is, to glew or soder the lips of the wound together, not procuring matter or corruption to it, as is commonly seene in the healing of wounds.*" Historie of plants, folio. Lond. 1633. p. 360. Who was the discoverer of healing wounds by this simple and rational process is not of much importance. Gerard certainly has the honour of having employed it in the darkness and ignorance of the 16th century, (the 1st edition of the Historie of Plants was in 1597,) of understanding its principle, and using even the same term employed in modern surgery. He certainly did not learn the art of curing wounds by the *first intention* from Taliacotius, for his work, " *de curtorum Chirurgia per insitionem, &c.*" was but just published at Venice; the princeps edition, folio, bearing the date 1597. With the above passage extant in *Gerard*, no surgeon of a subsequent period can fairly claim the discovery. The author of the " *Triall of Tobacco*," is so enamoured of Master John Gerard's balsam, that he considers it to have an excellence surpassing all others. After inserting the recipe, he adds, from Gerard, " I send this jewell to you women of all sorts, especially to such as cure and helpe the poor and impotent of your countrey without reward. But unto the beggarly rabble of witches, charmers, imposters, and such like cozeners, that regard more to get monie, than to help for charitie, I wish this medicine farre from their understanding, and from those deceivers, whom I wish to be ignorant herein." He entirely overlooks, as many others did, the efficient point so clearly stated by Gerard, " *that is, to glew or soder the lips of the wound together, not procuring matter or corruption to it.*"

* In two cases of stricture in the urethra, Dr. Shaw of Philadelphia, employed bougies medicated with Tobacco. Both cases appear to have received permanent cures. In one case, a thin smooth leaf of strong Tobacco, (*N. Tabac.*) previously moistened with water, was wrapped around a small sized bougie, and carried down to the stricture,

In the topical application of this plant the practitioner must carefully watch for the effects it may produce on the general system. Disturbance in the first passages, torpor of the brain, and a deathlike syncope, seem to have arisen from its application in the form of poultice or ointment. Where the cutis is exposed by a removal of the cuticle, or in extensive ulceration, this is most likely to happen. From its application to the *scrobiculus cordis*, where the cuticle has been whole, these symptoms have occurred.* To the varied forms

---

ture, where it was kept fifteen minutes, when it passed the stricture and went into the bladder. At this time the patient complained of sickness, and perspiration appeared on the forehead. As soon as the bougie was withdrawn, he discharged half a pint of urine, which flowed in a natural stream. The application of the bougie was repeated twice a day for three days, and the patient was cured. In another similar case the bougie was smeared over with an extract of Tobacco. A sensation of sickness and debility also took place in this case, while the bougie remained in the urethra. The operation was repeated three times, and produced such torpor and relaxation of the sphincter vesicæ, as to render the patient unable to retain his urine. This inconvenience was removed by T. Lyttæ. These cases are sufficiently marked to raise an expectation of great utility from the employment of bougies medicated with Tobacco. *In those deplorable suppressions of urine, where every effort is unavailing to pass an instrument into the bladder by the urethra, is it justifiable ever to proceed to the desperate operation of puncturing the bladder, without first trying the effect of the* TOBACCO BOUGIE ?

* Its property of occasioning severe vomiting, when applied in the form of cataplasm to the region of the stomach, though an inconvenience in some instances, presents a powerful resource in others. In that torpor of the stomach occasioned by an over dose of opium, or in any instance where it is desirable to evacuate its contents, the organ itself being insensible to the stimulus of emetic substances taken into it, or the power of deglutition being lost, may some good be expected from the Tobacco cataplasm ? In cases of asphyxia, and, perhaps, apoplexy, where bringing the stomach into action is to be considered as the most powerful means of exciting the system, may not the same application be employed ? The injection, whether of smoke or decoction, is certainly less manageable. The cataplasm can be instantly removed, but the practitioner must submit to the frightful energy of the enema. Even the external application of this potent substance requires careful attention. A middle-aged countryman and his wife applied an infusion of Tobacco to the skin for the cure of the itch. In an hour they felt as if intoxicated with spirits. This was followed by violent head-ach, dry hot skin, excessive vomiting and purging ; spasmodic contractions of the hands and arms, and considerable dyspnæa. These symptoms continued as long as the solution of Tobacco remained on the skin. The warm bath speedily removed the derangement. Med. Comment Edin. xi. 327.

of of cutaneous diseases Tobacco has been applied either as a lotion or an unguent; even modern practice does not reject it in Tinea Capitis. Until the introduction of the quicksilver ointment superseded it, a decoction of Tobacco was the remedy used by shepherds for that contagious disease in sheep called the scab.*

The external application of Tobacco has not been confined to the cure of local affections. It has been thus employed with a view to the removal of some diseases of the system over which the *materia medica* has generally had but little influence. Epilepsy and general convulsions have been cured by the application of Tobacco in cataplasm and injection. For the verification of this we fortunately have not to look into the records of remote and credulous ages. The most enlightened physician of the present age, the late Dr. James Curry, has left this evidence of its medicinal powers.† " In a case of epilepsy, which occurred in the hospital practice, the paroxysm returned periodically every afternoon. In this instance a cure was effected by a cataplasm, formed chiefly of Tobacco, to the scrobiculus cordis, about half an hour before the expected return, by which a powerful impression on the system was produced, and the paroxysm of epilepsy prevented. This practice, repeated several days at the expected periods, probably destroyed the diseased association, for the cure was permanent. I was induced to use Tobacco on this occasion from having observed it to succeed in the cure of obstinate intermittents, when appied in the same manner, previous to the accession of the paroxysm." " In two recent cases," Dr. Curry adds, " I have employed the remedyin a different manner. Each of the patients laboured under general convulsion, and the paroxysm had returned so fre-

---

* The external application of Tobacco makes a great figure in the first writers on this plant. Magnenus has a section of his *Exercitatio* xiv. entitled, " *Morbi particulares externi quibus Tabacum subvenit.*" The subdivisions of this section are, 1. Ad ulcera omnia. 2. Ad pediculas, phtyriasim et tineam. 3. Ad porriginem, achores, et polypum. 4. Ad ranulam. 5. Ad strumas, tumores thymum. 6. Ad œdema, gangrenam, cancrum, lupum fistulasque. 7. Ad hæmorrhoides, et herniam. 8. Ad verrucas, clavos, cirones, perniones, lapsos ungues. 9. Ad vulnera omnia. 10. Ad morsum canis rabidi, ictus urticæ, punctiones, venenatosque morsus. 11. Ad mures, glires, cimices, pulmones marinos, &c. 12. Ad inflationes ventris, pruritus, ambusta, scabiem, pustulasque et furunculos. To this formidable catalogue may be added its honorable station in the *Ars Cosmetica*, ad ruborem faciei, dentium nigredinem, cutis asperitatem, VULTUSQUE TRISTITIAM.

† Medical Reports, Vol. I. 163.

quently quently, as to produce continued coma, and in the bye-standers an absolute despair of recovery. I ordered a decoction of half a drachm of Tobacco in four ounces of water, to be thrown up as an enema. This powerful agent penetrated the system to its very centre, roused the sensibility which the effusion of cold water and other external impressions could not awake; excited sickness, vomiting, and profuse perspiration, and interrupted the convulsive actions, which have never since returned. In each of these cases the recovery was altogether unexpected."*

The Tobacco ENEMA, whether in the form of smoke or decoction, is undoubtedly one of the most powerful agents of the *Materia Medica.* Its operation is sudden, violent, and in many instances, effectual.† This mode of employing the Nicotiana was early known to writers on the subject. Magnenus recommends *clyster tabaci*,‡ for colics, flatulence, in-

---

* In the "TRIALL OF TOBACCO," a thin 4to. written in the early part of the 17th century, by *Edmund Gardiner*, the author asserts that "the suffumigation of Tobacco being taken, is a good medicine for the starknesse or stiffnesse of the neck, called *Tetanus*, and for any pains or ACHES in the bodie, proceeding of the cause that Tetanus doth." Fol. 25. When externally applied, especially the Oleum Tabaci, to the neck and spine, the old writers had much confidence in it as a remedy for Tetanus. Vid. Magnenus and Neander.

† The author of the "*Use and Abuse of Tobacco*," 8vo. 1720, has this ridiculous direction for the management of the patient after the injection of the Tobacco Enema. "I have known it used," he observes, "with very good success, by making a decoction of it in *urine*, for a glyster, in a violent iliac passion, when several other things failed. The method was this; after having, with much difficulty, injected the glyster, and spread a carpet upon the ground, the patient was constantly rolled upon the floor for some considerable time, till he felt a strong motion for stool; at which time there was a copious discharge of hard excrements and wind, to the sudden relief of the tormented patient, and the joy of his despairing friends."

‡ MAGNENUS has this curious formula for the Tobacco Enema. R. Fol. Tabac. m. j. fiat decoctio in jure pulli gallinacei pinguis q. s. R. Hujus decocti ℥ix. Succi tabacini, sacch. rubei āā ℥ss. Mell. violati, olei communis āā ℥ij. fiat clysteri. There cannot be a more striking instance of the complex absurdity of the extemporaneous formulæ of this period than is seen in the following recipe for a Tobacco Enema. Take Mercury (*Mercurialis perennis*, a plant which has manifested some deleterious properties, and is one of our indigenous vegetables which should not be overlooked), rue, marshmallow, little centaury, each six handfuls; Tobacco leaves six; root of marshmallow ℥ss; linseed and fenugreck each ʒiij. cummin and anniseed each ʒiss. boil in a sufficient quantity of water, until one-third part be consumed; strain durated fæces, nephritic complaints, &c.; and Everhard recommends the enema in ileus. In the practice of modern surgery the value of the Tobacco Enema is fully appreciated. In every species and degree of hernia, where medicine is required, it may be serviceable; but especially in that alarming and dangerous state when the protruded parts become inflamed and painful, and cannot be returned. In the *Zoonomia* there is a case of hæmorrhage from the rectum cured by an injection of tobacco-smoke.* Neither must it be forgot-

---

strain lbj. add hiera picra ℥ss.; benedicta laxativa ʒij.; fresh butter and honey of roses each ℥iss. oil of rue and dill each ʒiss.; common salt ʒj. These are to be mixed to make an enema, the only efficient material of which is the Tobacco. The formula of Mr. Pott presents an admirable contrast. Pour a pint of boiling water on a drachm of Tobacco, is the plain recipe for the Tobacco enema of this celebrated surgeon.

* "Mrs. ——— had for twelve or fifteen years, at intervals of a year or less, a bleeding from the rectum without pain; which, however, stopped spontaneously when she became weakened, or by the use of injections of brandy and water. Lately the bleeding continued above two months, in the quantity of many ounces in the day, till she became pale and feeble to an alarming degree. Injections of solutions of lead, of bark, and salt of steel, and of turpentine, with some internal astringents, and opiates, were used in vain. An injection of the smoke of Tobacco, with ten grains of opium mixed with the Tobacco, was used, but without effect the two first times on account of the imperfection of the machine; on the third time it produced great sickness, and vertigo, and nearly a fainting fit; from which time the blood entirely stopped." Zoonomia, ii. 69. In a pamphlet published in 1720, on the "Use and Abuse of Tobacco," there is an instance recorded of this plant suppressing a hæmorrhage from the rectum. A leaf of Tobacco was thrust up the anus, and continued there some time: by this the discharge of blood was completely restrained. This case may suggest the propriety of employing the Tobacco Enema in other instances of hæmorrhage. So powerful are the effects of Tobacco, that whenever or however it is used, great caution is required. Those who have seen either the smoke or infusion thrown into the rectum, will not forget the symptoms of its potency. Perhaps some unfortunate events have followed the careless or improper use of this remedy. Fuller, who seldom wrote or spoke with reserve, hints at an unfortunate case where an unknown glyster, he conjectures of tobacco, was given. In the same paragraph he asserts, that one Mr. Osbeston, after an Enema of Tobacco infused in sack for a colic, "fell presently into horrid burning pains, convulsions, faintings, and so perished miserably upon the spot, as it were all in flames." And he cites from *Ettmuller*, "Clyster ex decocti Tabaci summe periculosus est, cum usum ejus (subitò ac modò applicatus fuerit) præcordiorum anxietates, lipothymias, vomitus, sudores circa frontem frigidos, totius feralem quasi pallorem, aliaque symptomata insecuta fuisse noverim."

 ten, ten, that in cases of asphyxia,* it has been employed to rouse the torpid system into action by the medium of the intestinal canal.

As the Tobacco injection produced such marked effects when thrown into the intestines, and was known to be destructive to insect life in many instances, the probability of its proving an active anthelmintic was an obvious conclusion. Many instances of its beneficial effects in cases of intestinal vermes are related, and also as the means of dislodging the larvæ of flies from other parts of the body. In obstinate cases of tape-worm, and where the patients are robust adults, this active remedy may, possibly, be used without hazard; but in children, who are the subjects most liable to vermes, it will be advisable to act with great caution, or, perhaps, to

---

* M. Desgranges, M. D. has enquired, with much research, into the use of Tobacco-smoke Enema in different species of asphyxia, particularly that of submersion, and in the treatment of many other diseases. His work is extremely valuable in that part of it which treats on the various kinds of asphyxia. *Journ. de Medicine*, 1791. The old writers on this plant seem to have been aware that it had the property of curing diseases in the lower part of the intestinal canal. "The powder of tobacco alone, or mingled with other lenitives, is a present remedy for the *Emrods*: for it perfectly cures them." Panacea, 29.—Vitet, who is a determined enemy to Tobacco, is compelled to allow that—La fumigation des feuilles introduite dans l'anus, calme les coliques venteuses, convient dans l'apoplexie pituiteuse, la léthargie pituiteuse, l'asphixie hystèriqne, l'asphixie par les passions de l'ame, l'asphixie des noyés, la tympanite sans inflammation ni disposition inflammatoire; et favorise l'expulsion des matières fecales. L'infusion des feuilles en lavement, est indiquée dans les mêmes especes des maladies, lorsque la fumigation n'a été d'aucun secours; elle produit une évacuation, beaucoup plus abondante des matiere sfecales, elle irrite d'avantage l'intestin rectum. *Pharmacopée Medico-Chirurgicale*, 4*to a Lyon.* 1783. p. 198.—The powers of the *Tobacco Enema* are so remarkable, that they have arrested the attention of practitioners in a remarkable manner. Of the effects and the method of exhibiting the smoke of Tobacco *per anum*, much has been written. Beside the writers of our own country, I refer to Rozier, *Observations sur la Physique*.—Jean-Andre Stisser, *de Machinis fumiductoris curiosis*, *sive*, *fumum impellende intra corpus instrumentis*, *eorumque in praxi Medica adhibendi ratione et usu*. 4*to*. *Hamb*. 1686.—Schaeffer, *Gebrauch und Nutzen des Tobacksraus-clystier*.—De Haen, *Rat. Med*.—Gaubius, *Adversariorum varii argumenti*. 4*to*. *Leyd*. 1771.—Jos. Jacquer Gardane, *Avis au peuple sur les Asphyxies*. *Paris*. 12*mo*. 1774.—Feller, *De enematibus*, *atque nova*, *fumum Tabaci inferendi*, *methodo*. *Leips*. 1781. —Pin, *sur le succès de l'établissement à Paris en faveur des personnes noyées*.

reject reject it altogether, except where there may be peculiar and urgent danger from the disease.*

From the internal use of Tobacco more has been expected than any individual remedy will ever perform. Apoplexy, asthma, consumption, dropsy, rheumatism, and gout, have submitted to its powers, if we can believe the records of medicine.

That Tobacco has been employed for various affections of the brain is not to be denied. In apoplexy, coma, vertigo, and epilepsia, it was once thought to be a remedy of importance; but in the shifting of medical opinions, it has at length become more suspected of producing this class of diseases, than believed to cure them. We have, however, a well-authenticated instance of its curative powers in epilepsy and convulsions, (vid. Curry); and one of the most learned and judicious physicians of the latter end of the 17th century, says, "though I know many have an opinion of its (Tobacco) being narcotic, or otherwise injurious to the brain, and consequently disposing to apoplexies; yet (to say nothing of my having used it, and not sparingly, for many years, without finding any such effect of it) the very common custom of taking it for so many scores of years since it began to be in vogue, must have made such a quality, if it had it, evidently taken notice of; and consequently common prudence would have obliged people to have left it off long ago, as deleterious, if experience did not evidence the contrary: for there is no man but if, laying aside prejudices, he will give himself the trouble to observe, may easily find, that very many live to great years, and in as great a state of health as those who take it not, that have long used it, even immoderately. My sense of it is, that in those persons with whom it is found to agree, 'tis a very good drainer of humours, and may supply the place of fontanels, or at least that fewer of these may be necessary to those who abound with moisture. But there seems another reason why Tobacco may be useful to those who are disposed to apoplexy (under a supposition of its agreeableness), viz. that by reason of the vellication the

---

* The juice of the leaves of Tobacco clarified, and with sugar made into a syrup, and taken in a morning in a small quantity, drives forth stomach and belly worms; yet you must bruise the leaves and wrap them in a cloth, and lay them to the navel of the patient, and give him a glyster of sugar and milk. Panacea, p. 2. 29. *Magnenus* says nearly the same. De Tabaco Exertitationes, 247. A long string of authors might be quoted in evidence of the anthelmintic properties of Tobacco. It is, however, sufficiently certain, that it has vermifuge powers, but great caution is required in the employment of them.

smoke of it impresses on the nerves of the mouth, it makes them contract themselves, and so by consecution the whole brain comes to be analogously affected. So that if the brain happen to be more lax than ordinary, and thereby disposed to receive an afflux of blood and serum, as I take it to be, especially after a person has had and escaped one fit, as well indeed as in many other cases of preceding nerval indispositions, I see nothing but it may prove a very useful administration toward restoring the tone of it."*

Notwithstanding the evidence of men who daily smoke largely without inducing apoplectic symptoms, which Dr. Cole uses in support of his opinions on the effects of Tobacco, there are many facts to be produced of its having occasioned somnolency, and at length fatal *coma.*† But this species of dangerous operation of Tobacco, in direct opposition to Dr. Cole's conclusion, can only arise in systems accustomed to its use, and with which it appeared to agree when taken in moderate quantity. The brain is only endangered when use permits an excessive dose to be taken without disturbance, and allows its soporific principle to act silently on the sensorium. In those unaccustomed to its use, its operation is always on the *primæ viæ*; it then seldom manifests narcotic properties, but the whole frame is thrown into violent commotion. An attention to this double principle of action will, perhaps, enable practitioners to manage this plant with more success, and in very different trains of diseased action. When employed as a narcotic with a view to appease irritation, the dose must be small, and prepared in a particular

---

* *Physico-Medical Essay concerning the late frequency of Apoplexies. By* William Cole, *M. D.* 8*vo. Oxon.* 1689. It may be observed, *en passant*, that Dr. Cole deduces the increase of apoplexies and convulsions in 1684, from the continued intense cold of 1683.

† *Ephem. Nat. Cur. Dec.* 2, *Ann.* 10.—Hellwig, Obs. Phys. Med. give instances of this effect. Hellwig, in particular, has two instances of coma from smoking eighteen pipes of Tobacco. But a much larger quantity has been smoked without producing this deleterious effect. In the *Prax. Mayern.* there is recited an instance of the whimsical practice of *Dr. W. Butler*, about the year 1600. For a defluxion on the teeth, Dr. Butler directed a patient to smoke Tobacco without intermission until he had consumed an ounce of the herb. The man was accustomed to smoke, and took twenty-five pipes at a sitting. This first occasioned extreme sickness, and then a flux of saliva, which, with gradual abatement of the pain, ran off to the quantity of two quarts. The disorder was entirely cured, and did not return for seventeen years. The observation of Butler, " that a hard knot must be split by a hard wedge," shews that he considered this to be a desperate remedy.

way; way: * when intended to rouse the system from *asphyxia*, the mode of employing it is well understood. Tobacco has another degree or mode of action, intermediate between the preceding, partaking of both, but being exactly neither. When employed to relax spasm the quantity should be sufficient to excite nausea, debility, and the approach to syncope. If it is ever useful in apoplexy it is, when acting with the full energy of its stimulant powers, it rouses the brain from torpor through the medium of the stomach and intestinal canal. From observing in many cases, and perhaps in the greater number, that as soon as the stomach and bowels are acted upon, especially the stomach, persons come out of the apoplectic paroxysm; a conclusion is formed favourable to the employment of this remedy. If apoplexia is to be considered with Nosologists as a genus containing many species, this remedy can hardly be considered as equally proper in all of them. In *apoplexia sanguinea* it cannot be admissible until the vessels are emptied by the lancet. In *apoplexia-serosa*, *venenata*, *mentalis*, *cataleptica*, *suffocata*, *immersorum*, *hysterica*, *epileptica*, *verminosa*, *ischuriosus*, † there is reason to expect it may be serviceable, if managed with sufficient expertness. The *apoplexia hydrocephalica* is an hopeless case. In the apoplexy, or asphyxia, arising from narcotic poisons, (among these is tobacco itself) and from excessive drinking of spirits, it seems peculiarly proper either in enema, or in cataplasm to the *scrobiculus cordis*. In many of these cases it is impossible ‡ to get remedies into the stomach, the power of deglutition being lost: how valuable then is one of certain action, when applied externally, or thrown into the bowels *per anum*. In the apoplexy that arises from a deleterious dose of the narcotic poisons, and in the fatal asphyxia consequent to the excessive drinking of spirits, there is no vomiting. When vomiting does occur, persons recover, or

* A portion of the active part of Tobacco may be dissipated by long boiling; and we are of opinion that the preparation in extract, as prescribed in the Wirtenberg Dispensary, may be employed in pectoral cases with more advantage and safety than the simple infusion or decoction made by short boiling.—CULLEN, MAT. MED.

† It can hardly be necessary to recal the reader's attention to the cases of suppression of urine treated by Dr. Shaw, with the tobacco bougie.

‡ I am certainly aware that a tube may be passed by the æsophagus into the stomach, and that medicines may be conveyed by this means into that organ, when the power of swallowing is lost. But the application of the Nicotiana either to the bowels by enema, or in some form to the region of the stomach, seems far preferable.

have have a better chance to recover, from intoxication and the *apoplexia venenata.**

On the use of Tobacco in *Asthma* and consumption, particularly the smoke of it, the old writers are minutely laborious. They assert its efficacy with confidence, and cases are produced to support their assertion. The following, from Edmund Gardiner, is a favourable specimen; perhaps, its quaintness recommends it.

"I know myselfe a verie learned gentleman dwelling at Buckworth in Huntingdonshire, who had long languished of an orthopnoia; so that by reason of too many slimy and waterish humours which distilled downe from the braine into the chest, his lungs were so choaked, that hee could not breath, but by holding his necke upright, insomuch that many times, especially in the night season, he was in danger to be suffocated: by meanes of which, and his extreame cough, together with an extenuation of the whole bodie, he was adjudged *Tabidus* of most physicians that visited him, yea, and to be almost past all hope of recoverie; and one of the physicians being first asked his opinion, concerning the sicke patient, cast out these words:

Virtus lassa cadit, solvuntur frigore membra,
Vitaque tartareas fugit indignata per umbras.

And because I being his familiar friend, and one that was best acquainted with his whole course, and order of physicke and diet, which was both rationally prescribed, and diligently taken and observed, yet nothing taking effect that was administered, when all men thought he would have died, he was at length counselled to take Tobacco in fume; which he daily did, and onely by this way by little and little, he recovered his former health of bodie. His friende before

* I have no inclination to enter on the controverted point of the employment of emetics in apoplexy; the connection I have mentioned to exist between the reversed action of the stomach and the subsidence of the fit, has been suggested by my own observation in a great variety of cases, and under very different circumstances. I have never seen a case of apoplexy or asphyxia, when full vomiting spontaneously arose, or was excited by art, that did not escape the immediate danger of the paroxysm. The obscure history of the reciprocal sympathies that operate from the brain to the stomach, and from the stomach to the brain, I leave to be unravelled by *profound* physiologists. To those who would see much ingenious, much learned, and much useful investigation, on the employment of emetics in apoplexy, I recommend the perusal of the controversy between Langslow and Crowfoot, as found in the preceding volumes of this Journal.

spoken spoken being a doctor of physick, and he who cast forth the former verses, seeing the sodaine mutation, and wondring at the good successe, he thus again pleasantly and conceitedly answered:

> Mors aderat, cymbamq; Charon remosq; parabat:

> Asseruit medicina senem iam ætate trementem,

> Restituitq; novas effæto in corpore vires.

"Smoaking Tobacco, says Dr. Bree, (certainly the most valuable writer on the subject of asthma) is practiced by some asthmatics, who mistake the great secretion of saliva for a necessary evacuation. I am satisfied that a much more copious determination of lymph is made to the bronchia and salivary glands by smoaking, and it will be entirely conformable to the rules of the animal economy, if the habit of such secretion be confirmed, though this exciting cause be absent. I have persuaded some asthmatics attacked with the first species *(convulsive asthma from the irritation of effused serum in the lungs)* to abandon the practice, with great advantage to their health. The system cannot obtain the necessary supply of oxygen sufficiently fast in this species of the disease, even if the disoxygenating property of tobacco fumes be not employed."

Much as the opinions of this physician, in all parts of medical science, deserve to be respected, and especially in every form of disordered respiration; we must not be led to reject, out of veneration for those opinions, tobacco fume in every difficulty of breathing, or disorder in the chest. He speaks here of its inutility in one species of asthma only. There are forms of *dyspnœa* which do not arise from mechanical obstructions, nor are occasioned by effused serum irritating the sensient fibrilla of nerves, but result from some disturbed or irregular sensibility not easily explained; in which no organic alteration, or abundant morbid secretion is observed. In these cases it is that the fume of tobacco and other narcotics has been serviceable by mitigating the paroxysm; and by obviating the effect, finally perhaps, so weaken the habitual morbid action, as to wear out the disease. Taken in this view, no reasoning *a priori* can convince that the smoking of tobacco may not be serviceable in some form or degree of asthma. It is obvious, however, that as it is capable of exasperating the complaint, its use will require the superintendance of an experienced physician.*

---

* *Neander* goes very minutely into the efficacy of tobacco in asthma. Tobacologia, 4to. Lugd. Bat. 1622. p. 36, gives many formulæ of the plant

In *Dropsy* Tobacco has been employed with more effect than in any other disease. At least the evidence of its efficacy is more distinct, and the history of its employment more rational, than is remarked in other instances.

The earliest writers on this subject were sufficiently impressed with an opinion of the powers of *Tobacco* in hydropic complaints; but their details are vague, and generally unauthenticated by actual observation.* At length an experimental inquiry into the powers of this substance on the kidnies, was undertaken by *Dr. Fowler;* and to him the faculty is indebted for a collection of facts which elucidate the extent and quality of its diuretic properties. This physician was so strongly influenced by a letter sent by Dr. Garden, of Charlston, South Carolina, to Dr. Hope, of Edinburgh, in 1775, stating the surprising effects of the *ashes of tobacco* † in hydropic cases, that he undertook an investigation of the subject; and in 1785, published ‡ the result of his inquiries.

---

plant as employed in this disease. Among these are syrups, powders, wines, waters, cerates, &c. As a specimen of the mode of prescribing the *Nicotiana* in the 16th century, I cite the following, *Syrupus optimus ad Hydropicos*. ℞. Foliorum sanæ sanctæ Indorum. m. vj.—Hyssopi sicci, Pulegii regalis, Ceterach (seu asplenii) ana M. jss.—Calamenti minoris, p. ij.—Sem. anisi, sem. urticæ, sem anethi, ana ʒiij.—Galangæ, hellebori albi, ana ʒiiiss.—Asari, agarici, ana ʒij.—Rad. angelicæ hort. R. iridis, costi, amomi, polipodii quercini, ana ℥j. These, in powder, are ordered to be infused in six pints of wine vinegar for three days in the sun, in a glass vessel; afterwards evaporate one half over a slow fire, strain and add mel rosarum, lbj. sacchari lbss. Boil again, *to the consumption of the vinegar*, and aromatize with saffron, ginger, and mace, ana ℈ij.

* Everard, Gardiner, Neander, and many others, were fully, if not rationally, convinced of its efficacy in hydropic effusions.

† "Here we use with surprisingly great efficacy, in dropsical cases, the alkaline fixed salts of tobacco. It is given from the quantity of half a dram to a whole dram, twice a-day, in as little liquid as possible. During its operation, also, the patient is enjoined to use very little liquid and much exercise. This remedy proves deobstruent, diuretic, and purgative, *and does make great cures*. After it, however, I generally give purges of rhubarb and sal martis every second or third day, according to circumstances. Besides this, I sometimes use a strong decoction of the radix scillæ, with some syrupus scilliticus, and two, three, or four grains of kermes mineral or tartar emetic. These I find a most efficacious purgative and diuretic." *Extract of a Letter from Dr. Garden. Med. Comment. V.* iii. 330.

‡ Medical Reports of the Effects of Tobacco, principally with regard to its diuretic Quality in the Cure of Dropsies and Dysuries: together with some Observations on the Use of Glysters of Tobacco, in the Treatment of the Colic. By Thomas Fowler, M. D. 8vo. Lond.

Dr.

From this it appeared that Tobacco might be exhibited both as *a safe and efficacious diuretic* in cases of dropsy and dysury. In thirty-one dropsical cases in which Dr. Fowler employed it, eighteen were cured and ten relieved. In eighteen cases of dysury ten were completely cured, and seven relieved. Since the period of Dr. Fowler's publication, many instances have occurred of its efficacious operation in *anasarca*, in *ascites abdominalis*, and in *hydrops ovarium*. The ingenious Dr. Ferriar often found it to be a ready diuretic.* The fashion for using it as a diuretic has unwisely

---

Dr. Fowler gave the *Nicotiana* in various forms. He employed it chiefly in infusion, but thinks it may be used with advantage in a spirituous or vinous tincture, in an *acetum nicotianæ*, and also in pills. A pound of each of the preceding menstrua with an ounce of the leaves of the *N. Tabacum*, are the quantities he employs, for the tinctures and acetum. For the pills he gives the subsequent formula.

R. Pulveris foliorum nicotianæ Virginiensis cautè siccatorum.
Conservæ rosarum rubrarum, utriusque drachmam unam.
Mucilaginis gummi Arabici, quantum satis sit. Misce, fiat massa de qua pilulæ sexaginta formantur.

This he considers as one of the most certain modes of exhibiting the Nicotiana, and observes, that upon several trials he found these pills to be powerfully diuretic.

* The latest and the best medical Dictionary in the English language, compiled by Bartholomew Parr, M. D. of Exeter, (we have now to lament the loss of this most industrious and ingenious man, who died a few weeks since) says of the *N. Tabacum*, that "its emetic power prevents its acting as a laxative, except in glysters; and as a diuretic, except in the form of its alkali after burning. The oil which remains adhering to the salts, adds to the diuretic power of the alkali, *and it has been supposed useful in dropsy*. In the days of its fashion we used it, but without such decided success as to continue it." *Art. N. Americana*. This is an imperfect account of its powers, and incorrect, inasmuch as Dr. Fowler found an infusion of the plant to be the most convenient and efficacious form for its exhibition. Under *Ascites* Dr. Parr does some justice to the *Nicotiana*; for he says, "it appears to be an active, useful medicine, *meriting much more attention than it has received*." He still seems to mistake when he dwells on its alkaline salt as the form preferred for its diuretic properties, by Dr. Fowler.

It would be a violation of candour to suppress the opinion of Cullen. "The infusion of tobacco when it is carried into the blood-vessels, has sometimes shewn its stimulant powers exerted in the kidnies; and very lately we have had it recommended to us as a powerful diuretic of great service in dropsy. Upon the faith of these recommendations we have now employed this remedy in various cases of dropsy, but with very little success. From the small doses that are proper to begin with, we have hardly observed any diuretic effects; and though from larger doses they have in some measure appeared, we have seldom found them considera-

 ble:

passed away, and affords an impressive instance of disappointed expectation, succeeded by injudicious neglect.

MODERN science knows nothing of the employment of Tobacco in Gout; we must apply to the uncertain and equivocal records of the first writers on the subject, for the antipodagric qualities of this plant.* Everard speaks very positively of its possessing such qualities, and that it even radically cures the disease, when taken in the form of snuff. Its external application either as cataplasm or ointment was esteemed to quiet the painful part of the arthritic paroxysm. The *extractum peticum* of some of the old Pharmacopœias was believed to be a powerful remedy in jaundice, dropsy, asthma, and gout; and that it was almost a specific in tertian and quartan agues. The violent operation of Tobacco may possibly point it out as a medicine likely to remove chronic affections not depending on organic alterations in the vital parts; and especially as affording a probable means of removing the gouty diathesis. It is evident that the fact of its acting with great and unexpected violence will demand the most cautious employment, and a minute investigation of the peculiarities of the patient.

IF a detailed examination of all the diseases in which tobacco has been used, were to be given, with the reasons for its employment, and the rationale of its action, as founded on the shifting hypotheses of different periods, these remarks would swell into a volume. It may not be deemed improper, however, before the subject is dismissed, to add some observations, conjectural indeed or analogical, on the probable effect this *potent vegetable* might have on a disease, the frightful symptoms of which are only to be paralleled by its unvarying fatality.

---

ble: and when to obtain these in a greater degree, we have gone on increasing the doses, we have been constantly restrained by the severe sickness at stomach, and even vomiting, which they have occasioned: so that we have not yet learned the administration of this remedy so as to render it a certain or convenient remedy in any cases of dropsy." *Materia Medica.* GERARD mentions a curious fact: "A strong country man having a dropsie, tooke four ounces of the juice of tobacco, and having waked out of his sleepe, called for meate and drinke, and after that became perfectly whole." In this instance it operated upwards and downwards; the patient exhausted fell into a deep sleep, and awaked perfectly cured.

* If the *Eau medicinale* were proved to be a composition made from tobacco (vid. this Journal, vol. xxiv. p. 353) it would shed a temporary brilliance on the genus Nicotiana. Temporary, however, it would only be, for this wonder-working *nostrum* is following its brethren, with rapid strides, to the tomb of the Capulets.

The

The disease produced by the bite of a *rabid* animal, denominated improperly, perhaps, *hydrophobia*, as far as I know, is always fatal. Stimulant, tonic, narcotic, and antispasmodic remedies have been employed in every shape: but in whatever form or quantity they have been had recourse to, they have disappointed the prescriber. Among the expedients which have been employed to arrest the progress of HYDROPHOBIA, I am not aware that Tobacco has been used to the maximum of its powers.

This disease seems to consist of a series of spasms, readily excited by many trivial circumstances. Attempts to swallow drink, the sight and perhaps the thought of cold water, and in a degree of all fluids whether heated or cold, the sound of water dashed on the ground, a current of cool air, the appearance of polished and reflecting surfaces, the sudden shutting of a door or the waving of a bed curtain, (perhaps as putting the air in motion), all excite the paroxysm; which recurs at short intervals, until the patient is exhausted. A highly morbid sensibility, an extraordinary increase of irritability, or possibly a combination of these, reciprocally acting upon and exciting each other, appear to constitute the *hydrophobia rabiosa.*

The property that the whole of the genus *Nicotiana* possesses, but peculiarly in one of its species, *N. tabacum*, of resolving the spasm of muscular fibre, mark it to be a remedy decidedly proper in a disease, the essence of which is a series of spasmodic actions.

The failure of every means hitherto used to overcome this disorder would justify the employment of Tobacco, did not a knowledge of the nature of the disease and of the qualities of the plant afford a rational prospect of advantage.

In the management of the *H. rabiosa* an impediment arises from the difficulty, and in some of its stages, from the impossibility of giving medicine. In this dilemma the Tobacco presents a peculiar facility. Its power over the system, when injected in fume or decoction, into the intestinal canal *per anum*, or, when the prepared plant is applied in the form of cataplasm to the *scrobiculus cordis*, is well known.

I am not sanguine enough to be convinced that the *Nicotiana* will prevent the hitherto invariably fatal termination of this disease; but I am convinced, from its manifest actions on the animal fibre, and on the functions, that it may be prescribed on more rational grounds than many remedies which have been employed in the HYDROPHOBIA RABIOSA.

THESE "Remarks" will probably afford ample evidence, that no vegetable substance has more excited the attention of

 mankind mankind than *Tobacco*. That its properties are of a character to demand attention, also appear to be incontrovertible.

When employed as a luxury, it possesses a peculiarly soothing quality; it relieves corporeal fatigue, and it calms mental perturbation. In a ratio agreeing with these fascinating powers, it becomes dangerous, however, from the hazard of its abuse. When used in moderation, especially when smoked, no evil arises; on the contrary it produces quiet and equanimity, without benumbing the imagination, or weakening the judgment. When used to excess, either in the form of snuff or smoked, the corporeal faculties of taste and smell loose their acuteness of sensibility; and sottishness, dyspepsia, torpor, coma, apoplexy are often the final result.

Considered as an Article of the *Materia Medica*, it requires to be looked upon with prudent suspicion; but when managed with caution and judgment, it is,

1st. A ready evacuant of the first passages, and often an active diuretic.

2d. A soothing sedative, used in a particular form and appropriate quantity, in some morbid sensibilities of the nervous system, especially as occurring in the trachea and its bronchial ramifications.

3d. An effectual resolvent of local spasm when topically applied, as in stricture of the urethra.

4th. A powerful stimulant, possessing possibly some specific property, "capable of penetrating the system to its centre; rousing the brain from torpor; and by exciting new and extraordinary actions, breaking the chain of morbid habits and associations."

MEDICUS.

*February* 10, 1811.

